# Waveguide Manufacturing Technologies for Next-Generation Millimeter-Wave Antennas

**DOI:** 10.3390/mi12121565

**Published:** 2021-12-16

**Authors:** Lucas Polo-López, Pablo Sanchez-Olivares, Eduardo García-Marín, Jorge A. Ruiz-Cruz, Juan Córcoles, José L. Masa-Campos, José R. Montejo-Garai, Jesús M. Rebollar

**Affiliations:** 1 Group of RadioFrequency: Circuits and Systems (RFCAS), Escuela Politécnica Superior, Universidad Autónoma de Madrid, 28049 Madrid, Spain; Lucas.Polo-Lopez@insa-rennes.fr (L.P.-L.); pablo.sanchezo@upm.es (P.S.-O.); eduardo.garciam@uam.es (E.G.-M.); juan.corcoles@uam.es (J.C.); joseluis.masa@uam.es (J.L.M.-C.); 2 Institut d’Electronique et des Technologies du numéRique (IETR), INSA, 35700 Rennes, France; 3 Information Processing and Telecommunications Center, Universidad Politécnica de Madrid, 28040 Madrid, Spain; joseramon.montejo@upm.es (J.R.M.-G.); jesusmaria.rebollar@upm.es (J.M.R.)

**Keywords:** millimeter-wave devices, waveguide manufacturing by subtractive machining, waveguide manufacturing by direct metal laser sintering, waveguide manufacturing by stereolithography

## Abstract

Some recent waveguide-based antennas are presented in this paper, designed for the next generation of communication systems operating at the millimeter-wave band. The presented prototypes have been conceived to be manufactured using different state-of-the-art techniques, involving subtractive and additive approaches. All the designs have used the latest developments in the field of manufacturing to guarantee the required accuracy for operation at millimeter-wave frequencies, where tolerances are extremely tight. Different designs will be presented, including a monopulse antenna combining a comparator network, a mode converter, and a spline profile horn; a tunable phase shifter that is integrated into an array to implement reconfigurability of the main lobe direction; and a conformal array antenna. These prototypes were manufactured by diverse approaches taking into account the waveguide configuration, combining parts with high-precision milling, electrical discharge machining, direct metal laser sintering, or stereolithography with spray metallization, showing very competitive performances at the millimeter-wave band till 40 GHz.

## 1. Introduction

The development of new wireless systems in the millimeter-wave band for diverse applications such as satellite communications, radar systems, or the different scenarios foreseen for the fifth-generation wireless technology radio [1,2], creates an increasing demand for novel antennas with very challenging requirements for their compactness, weight, and radiofrequency performance. The development of the high-frequency circuits and antennas for these emergent systems requires appropriate manufacturing techniques, which have advanced continuously during recent decades.

A clear example of this evolution is additive manufacturing (AM) [3,4]. AM has several advantages, such as the ability to use customized geometries with diverse materials. Previously, these properties have been successfully applied to microwave circuits [5,6,7,8,9,10,11]. Nevertheless, the use of AM at higher frequency bands is still under research, and tolerances still need to improve further to be more competitive. The goal of this paper is to show how different manufacturing technologies now coexist at the millimeter-wave band, where classical and modern approaches are combined to obtain optimum performance of circuits and antennas for the front-end of emergent high-frequency systems.

Printed circuit board (PCB) antennas have traditionally been the preferred solution for creating compact and lightweight antennas [12,13]. Recently, considerable effort has gone into the creation of PCB antennas with multi-function radiation characteristics [14,15,16]. However, due to relatively high-power losses associated with the use of dielectric elements, especially in planar transmission media, this technology is still far from being able to scale up to the frequencies required in the new millimeter wave systems.

In contrast, waveguide-based antennas [17,18,19] are a well-established technology that, due to their absence of dielectric parts, can work with high powers and exhibit low loss levels. Nevertheless, the approaches employed traditionally to manufacture these kinds of antennas, involving 3D metallic pipes, have limited the creation of designs with complex geometries at very high frequencies. It is clear that planar technologies provide easier routing than do 3D pipes, whose shape was traditionally rectangular or circular [20]. Nonetheless, the development of new manufacturing technologies during recent years has opened up the possibility of building waveguides and antennas with very complex geometries that could not be built using classical approaches. This paper presents several approaches for the manufacturing of these kinds of antennas, together with state-of-the-art examples of devices built using them.

First, high-precision subtractive machining is considered. Although this is a quite classical approach, it is possible to manufacture highly complex devices using it. The key idea is to segment the device into different parts that are built independently, to be assembled together subsequently. Although this approach may seem simple, the problems associated with segmentation involve a very close interaction between electrical and mechanical engineers. On one hand, from the electrical point of view, the need for more parts to be assembled implies more insertion losses and can reduce the power-handling capability of the device, while increasing the risk of passive intermodulation issues, as more flanges are needed to connect the different parts. On the other hand, a small number of parts may lead to very difficult features from the mechanical point of view, and tolerances may subsequently destroy electrical performance. This trade-off must be resolved by experienced practitioners. This paper will provide an example of a successful network implementation.

Next, direct metal laser sintering (DMLS) is presented. This technique enables the printing of 3D metallic objects by using a high-power laser to bind together powdered metal particles [21]. This technique has been used recently to develop antennas and other waveguide devices in millimeter-wave frequencies [22,23,24] up to W-band [25]. DMLS is quite new in the antenna field. As will be shown, it still presents certain limitations, but it has the great advantage of allowing the monolithic (i.e., single part) fabrication of certain waveguide devices that could not be manufactured using conventional subtractive techniques, while still obtaining the great mechanical robustness of 3D metallic manufacturing.

Finally, an approach based on stereolithography (SLA) manufacturing of the waveguide parts with a posterior metallization is presented for a conformal array. SLA is an additive manufacturing method based on UV curable resins that can provide a high-precision surface finish on plastic materials [26]. Therefore, a proper metallization technique is indispensable for SLA printing to become a cost-effective method of manufacturing hollow waveguide structures [27,28,29]. When working at high frequencies, special care must be taken in the post-metallization process. There can be problems with adhesion of the metal to the plastic. Therefore, this process must be well-controlled to obtain a high-quality surface finish. This approach can overcome certain roughness problems that appear in DMLS devices, while also producing lighter and less expensive devices. However, the lack of access to internal channels to metallize the part restricts the monolithic fabrication of some structures.

During recent years, the authors have worked on the manufacturing of several high-performance waveguide-based antennas using different manufacturing techniques, many of which belong to the additive manufacturing family. The present study presents the knowledge acquired during these years. It covers many manufacturing details and strategies that are key to the successful manufacturing of these devices, which details are not typically discussed in papers focused on the electrical design of the antennas.

## 2. High-Precision Subtractive Machining

Machining using high-accuracy subtractive means has been the classical approach for the manufacture of waveguide devices. Starting from the block of an electric conductor (typically aluminum or brass, although it is possible to use other metals with good electrical conductivity), the internal parts of the waveguides are carved out (typically by a drill, although, as will be discussed, other options are possible). These techniques normally provide a smooth finish for the internal walls of the waveguide, resulting in a low level of power losses. Additionally, as the waveguide device is built from a block of metal, it will demonstrate great mechanical robustness [20].

Moreover, subtractive manufacturing techniques provide the clear advantage of having been established for a long time; therefore, they present a high degree of reliability [30]. Although they typically require dividing the device into several parts and, in comparison to additive manufacturing, they are less flexible with respect to the geometries that can be built, these kinds of techniques should be chosen whenever possible.

The main disadvantage of subtractive manufacturing is that it normally requires the division of the device into several parts that are manufactured independently, for later assembling to create the desired structure. This assembling process has two critical points: the alignment and the fastening of the different parts.

A misalignment will alter the internal faces of the waveguides, which will affect the response of the device. To avoid this, it is necessary to introduce alignment pins into the design to guarantee proper positioning. Moreover, it is not only important that the number of pins is great enough, but also that they are placed at the appropriate positions in the structure.

The parts that comprise the device are joined by means of assembling screws, conveniently located. It is very important that these screws tighten the different parts with enough strength to avoid any power leakage, which would increase the power losses of the device [31].

**Figure 1:**
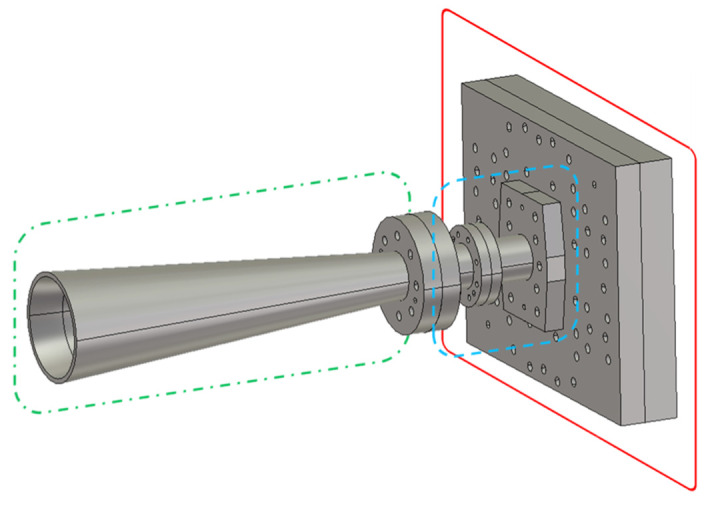
Monopulse horn antenna [22]. The three components that form the antenna are highlighted by different colors: solid red for the comparator network; dashed blue for the mode converter; and dashed-dotted green for the horn.

A recent work by the authors that illustrates the different possibilities offered by high-precision subtractive manufacturing can be found in [22], showing a novel monopulse horn antenna with a triple-radiation pattern. The antenna is comprised of three main components: the comparator network, the mode converter, and the horn itself [32]. A representation of the internal parts of this antenna is presented in Figure 1.

Each component of the antenna was built using a different subtractive machining technique. The horn was based on a splined profile, requiring higher accuracy than horns based on a simpler conical profile. It was manufactured using a high-precision lathe. Additionally, given the length of the horn (215 mm), it was of extreme importance to minimize any possible degradation of the symmetry of the component in its manufacturing.

**Figure 2:**
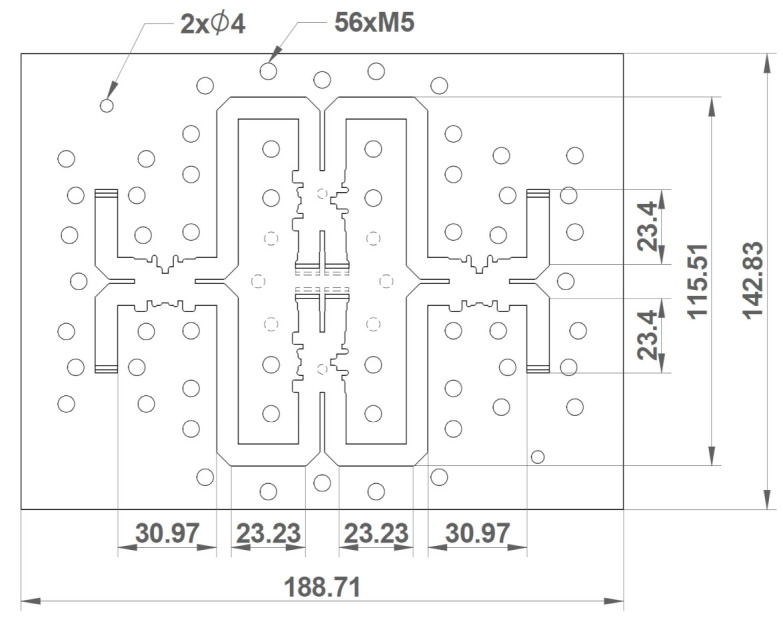
Top view of the comparator network. The rounding of the corners produced by the drill is included in the drawing, as well as the holes for inserting the screws and alignment pins. The main dimensions of the component, in millimeters, are annotated.

The comparator network was divided into two separated parts (i.e., the body with the routing and cover), which were built by means of using an H-milling plane along the direction of the width of the waveguides. Electrical discharge machining (EDM) was used for the waveguide sections corresponding to the ports at the external interfaces, as they were too long for the drill. A representation of this component, along with the main dimensions and the placements of the holes for the screws and alignment pins, is presented in Figure 2.

It must be noted that this milling process does not produce perfect right angles at the internal corners, but rather a certain rounding. Therefore, it was important to study the effect of this characteristic before manufacturing the device. Several simulations were carried out prior to manufacturing to assess the greatest drill diameter that could be used without producing a significant alteration of the device’s behavior. It was found that a drill diameter of 1.5 mm did not produce a significant alteration in the device’s performance.

**Figure 3:**
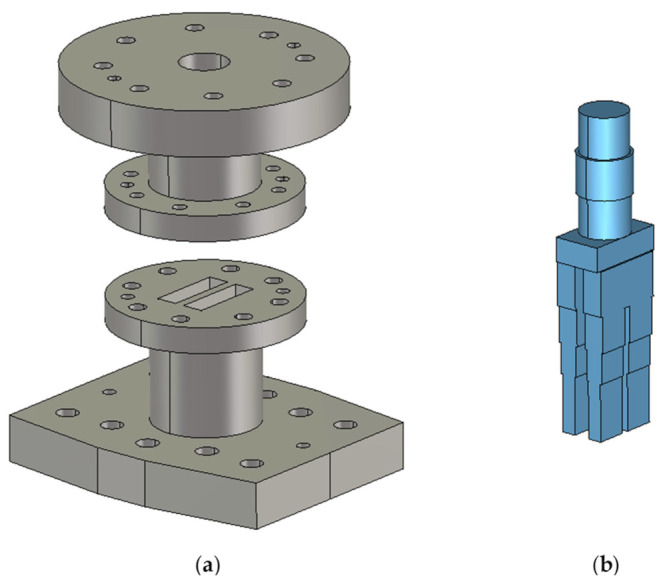
Representation of the mode converter: (**a**) the two waveguide parts that form the device, with the holes for screws and alignments pins visible; (**b**) depiction of the internal air cavities.

Finally, given the highly complex geometry of this device, EDM was used to build the mode converter in Figure 3. It should be noted that building all its internal cavities using conventional milling would have been impossible in practice. Moreover, it was necessary to divide the mode converter into two parts that were manufactured separately. In this way, EDM was able to adequately access the different waveguide sections. Figure 3 depicts these two parts, along with a representation of the internal cavities of the mode converter.

**Figure 4:**
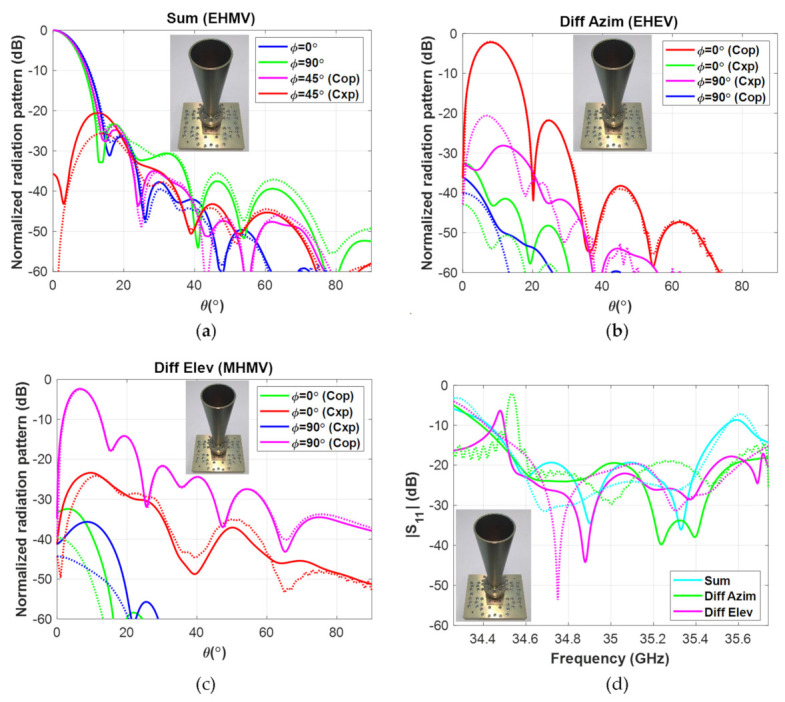
Measured (solid) and simulated (dotted) performances of the triple-radiation pattern monopulse horn antenna: (**a**) sum radiation pattern, $D_{irectivity,Meas}=24.85\;\mathrm{dBi}$ ; (**b**) difference in azimuth radiation pattern, $D_{irectivity,Meas}=22.66\;\mathrm{dBi}$ ; (**c**) difference in elevation radiation pattern, $D_{irectivity,Meas}=22.44\;\mathrm{dB}$ ; and (**d**) reflection coefficient for each radiation pattern.

As is shown in Figure 4, the measured performance of the final antenna matched, with great accuracy, the values predicted by the simulation. Hence, it can be concluded that, if executed carefully, subtractive manufacturing techniques constitute an accurate and reliable approach to manufacturing waveguide devices in the millimeter wave band.

The prototype in [33] has also been manufactured using high-precision subtractive machining. The main performance indicators, compared with the results obtained by [22], are summarized in Table 1. However, it is worth noting that both designs operate in different frequency bands, and for this reason a direct comparison was not evident.

## 3. Direct Metal Laser Sintering (DMLS)

**Figure 5:**
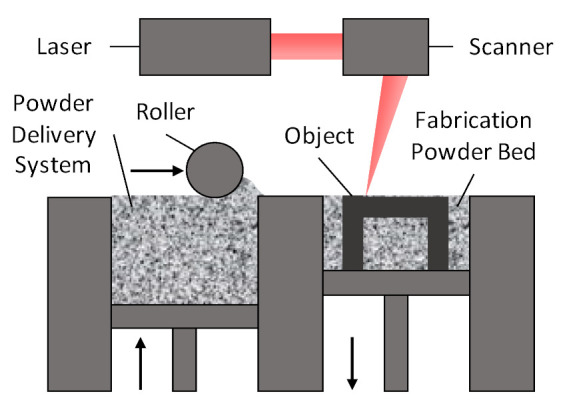
Schematic used generally to represent the direct metal laser sintering (DMLS) technique.

Direct metal laser sintering (DMLS) belongs to a family of additive manufacturing techniques known as selective laser sintering (SLS). SLS techniques are based on the successive deposition of layers of powdered material. For each layer, a high-power laser heats the regions that correspond to the object that must be built, melting the material to join the powder particles [3,34,35]. This process is illustrated in Figure 5. The unused material of a certain layer acts as support for successive layers, enabling the building of overhanging parts or even hollow structures, as long as they have some aperture that allows the evacuation of the powder after fabrication. This process opens up the possibility of monolithic (i.e., in a single part) manufacturing of waveguide devices, avoiding the problems associated with manufacturing in several parts, such as the alignment issues or power losses mentioned in the previous section.

In DMLS, a powdered metal alloy is used as the building material, and therefore the manufactured objects are completely metallic [21]. This technique is of special interest for the manufacturing of waveguide devices, as it avoids the need to perform a metallization of the built waveguide. The main disadvantage of DMLS is that the obtained parts exhibit a rough finish on their surfaces. This roughness, when present at the internal faces of a waveguide device, affects the device’s insertion losses. Nevertheless, DMLS is still a very young technology, and it is expected that it will evolve to achieve finer finishing in the near future.

A recent interesting work that illustrates the virtues of DMLS for the fabrication of waveguide devices can be found in [36], with a novel waveguide reconfigurable phase shifter (WRPS) adjusted by means of two tuning screws (TSs). Then, four of these phase shifters are integrated in a waveguide-fed phased array to provide reconfiguration capabilities. The phase shifters are used to adjust the relative phase differences between the radiating elements of the array, which allows control of the direction of the radiation pattern main lobe.

It must be noted that, due to the spacing constraints between the radiating elements of the array, it was necessary to build the four phase shifters in a single WRPS module. In this situation, it would have been extremely complicated to use a subtractive machining approach. Due to the reduced spacing between the shifters, there would not have been enough room to insert the required assembling screws and alignment pins. Here, additive manufacturing became the solution for overcoming the limitations of traditional manufacturing approaches.

**Figure 6:**
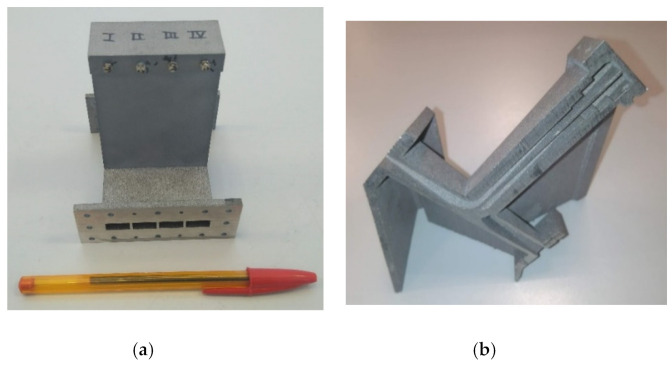
Photographs of the reconfigurable phase-shifters block built using DMLS: (**a**) frontal view; (**b**) cut view that allows observing the internal parts of the device.

As Figure 6 shows, the block of phase shifters has been built using DMLS. There are two reasons for choosing this technique over other additive manufacturing techniques based on printing the device in plastic and subsequently applying a metallic coating. First, the threaded holes needed to introduce the TSs would have been much more difficult to manufacture if the device were built in plastic. Although it is possible to incorporate threaded inserts into these kinds of designs, for the correct operation of this specific device a good electrical contact between the TSs and the waveguide walls is necessary, and would not have been be attained by the insert. In addition, the relatively elongated shape of the phase shifters might have posed certain issues during the metallization stage, causing some internal regions to be inadequately covered by the conductive layer. DMLS avoided these two issues as no metallization was required and the threaded holes for the TSs could be machined by conventional means after printing the device.

**Figure 7:**
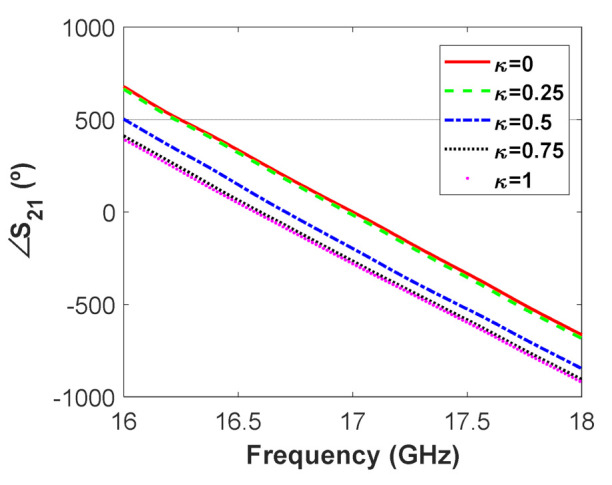
Measured phase shift produced by one of the WRPS for different values of TS (tuning screw) penetration.

Before inserting the WRPS module in the waveguide array, the performance of its phase shifters was studied individually, measuring experimentally their scattering parameters. Figure 7 represents the phase shift between the input and output ports of one of these phase shifters for different values of the parameter κ, which represents how much the TSs penetrate into the waveguide normalized to the waveguide height. It can be seen that screw penetration produces a change of the phase shift. Moreover, the phase response presents good linearity for the whole operation band (from 16 to 18 GHz). At the central frequency, the achieved phase shifting range is 270° which, as will be shown, is enough to scan the main lobe in the range $-25^{\circ }\leq \theta_{0}\leq 25^{\circ }$.

**Figure 8:**
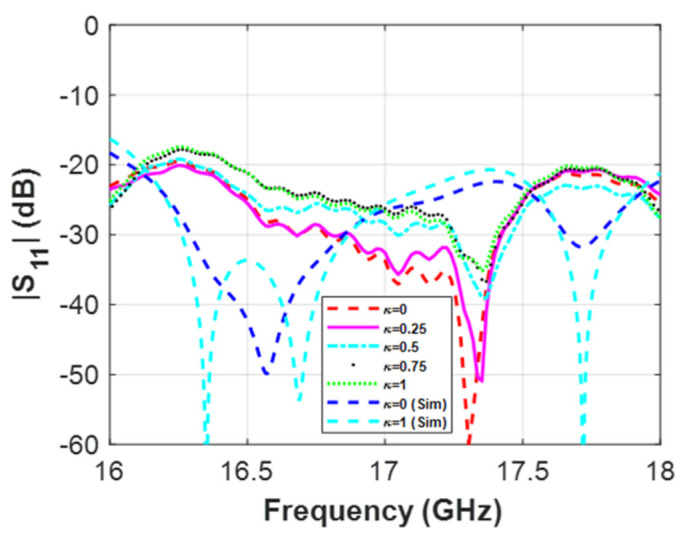
Measured and simulated (Sim) magnitude of the reflection coefficient at the input of one of the WRPS (the phase-shifter marked as I in Figure 6) for different values of TS (tuning screw) penetration.

Figure 8 presents the return loss at the input of one of the WRPS, which is better than 20 dB across the whole operation band for the different TS configurations. This level matches the predictions of the simulation, as shown by Figure 8.

**Figure 9:**
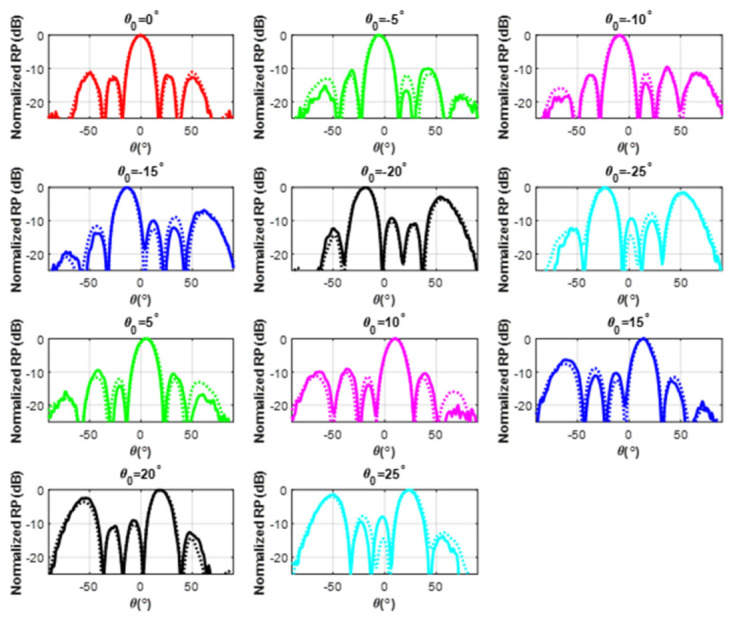
H-plane cut (ϕ=0) of the radiation pattern produced by the waveguide array for different scanning directions $\theta_{0}$. The solid line corresponds to the measurements and the dotted line corresponds to the simulations.

To demonstrate that the phase shifter provides scanning capabilities to the array, an experimental characterization of the radiation pattern was performed, scanning the main lobe in the range of $-25{}^{\circ}\leq \theta_{0}\leq 25{}^{\circ}$ with a step of 5°. To obtain the scanning for each of these directions, it was necessary that each shifter of the WRPS module introduced the phase shift presented in the right columns of Table 2. This table also presents the measured phase at each of the phase shifter outputs and, as can be appreciated, the obtained values are very close to the desired ones, which serves as an initial validation of the phase shifting characteristics of the proposed device. Moreover, Figure 9 presents the measured radiation patterns of each scanning direction in comparison with the simulated values. The match between both sets of data is very high, which again serves as a validation of the presented WRPS.

Reconfigurable phased arrays have been traditionally based on the use of tunable electronics to implement reconfiguration capabilities. For this reason, planar technology has been the preferred medium for manufacturing this kind of array, as it allows for easier integration of active parts. Nevertheless, there are some hybrid devices in which active components are integrated in a waveguide design, either with conventional waveguides [37] or substrate integrated waveguides [38]. Additionally, purely mechanically reconfigurable waveguide arrays have been achieved by using movable dielectric parts [39]. These two devices constitute an interesting frame of reference for comparison with the phased array in [36]. Table 3 summarizes the main performance indicators for each design. It should be observed that the reconfiguration mechanism of the three designs is very different. However, the lack of waveguide-based arrays with purely mechanical reconfiguration in the literature prevented further comparison.

## 4. Stereolithography (SLA) and Post-Metallization Process

An alternative that avoids the main drawback of DMLS (i.e., a rough finish of some internal surfaces) is provided by other technologies based on ultraviolet curable resins, such as stereolithography (SLA), which are based on the solidification of the resin layer-by-layer [26]. This technology allows the correct manufacturing of small elements and has low tolerances of approximately ± 50 μm. However, the prototypes manufactured with SLA require an additional plating process, which can be critical at high frequencies due to metal-to-plastic adhesion problems, an unacceptable metal surface roughness, or areas of poor metallization. Nevertheless, if metallization processes are well-controlled, reduced layer thicknesses (5–10 μm) with a smooth finish can be achieved.

**Figure 10:**
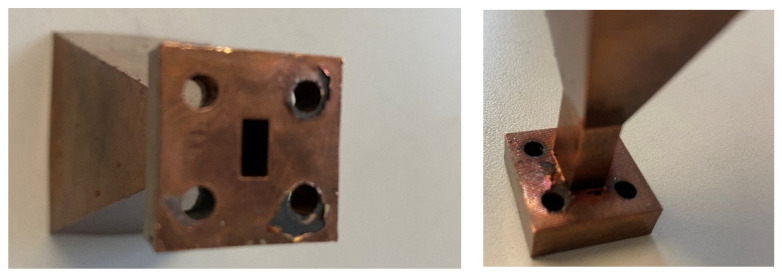
Initial prototypes manufactured in a commercial 3D printer and metallized with a copper coating.

Likewise, metal coating on plastics presents some mechanical deficiencies that cannot be obviated. This is a well-known technology, especially in the jewelry and souvenirs industries. Nevertheless, the metallic coatings used in these industries are poor metal alloys that cannot be used in microwave devices or antennas due to the conductivity required for efficient performance. On the other hand, the metal coating added to jewelry or souvenir pieces can present interesting mechanical properties that could be sufficient for some uses in the assembly process of RF devices. The key point is the preparation stage (prior to metallization) applied to the polymerized resin used in SLA plastic 3D printing, which can ensure a strong, uniform, and durable adhesion of the metal to the plastic. If this preparation stage of the polymerized resin is not properly executed, the external copper coating becomes fragile, crumbling and peeling like an eggshell. This effect can be seen in Figure 10, where two photos taken in our laboratory in a 3D printer show some initial prototypes manufactured with another companies’ external copper coating. 

Swissto12 [40] patented a copper metal plastic chemical coating that has been successfully used up to millimeter wave band [41,42,43,44,45,46]. This chemical process allows a homogeneous metallic coating to be obtained with a very low roughness (much lower than the typical roughness of DMLS technology). Thus, the combination of a high-precision stereolithography process with a subsequent low roughness metallization process allows the fabrication of passive devices and RF antennas at very high frequencies. For example, some designs of Ka-band antenna elements or microwave devices, such as a monolithic V-band OMT, were presented in [41]. All of them showed a high-quality response, as well as high agreement between the simulations and measurements. The transmission of W-band rectangular waveguides fabricated by SLA and metallized by the Swissto12 chemical process was shown in [42]. An effective surface roughness of a few hundred nanometers and an effective copper conductivity of 2 × 10^7^ S/m were estimated. Similarly, a Ka-band monolithic 2 × 2 horn array with a very complex topology was also presented with very satisfactory results. A reflection coefficient below −10 dB, good agreement between simulated and measured diagrams, and a difference between measured and simulated gain of less than 3 dB were obtained. Corrugated horn antennas at W-band were also presented in [46], showing 1 mm deep metallized corrugations with high-precision and satisfactory experimental results. Finally, experimental tests were carried out on waveguide designs and horn antennas on standard WR3.4 waveguide that offered good performance up to 300 GHz [45]. In some of these studies, the importance of the preparation stage to achieve good mechanical performance is highlighted (e.g., in the fixing of the screws at the waveguide flange). A disadvantage of this chemical process is the activation of the preliminary step, which makes it a relatively costly proceeding. The Swissto12 patented process contains the whole 3D printing plus copper coating operations, and is especially recommended for space or satellite applications, leading to medium-to-high costs if cellular deployment is intended.

**Figure 11:**
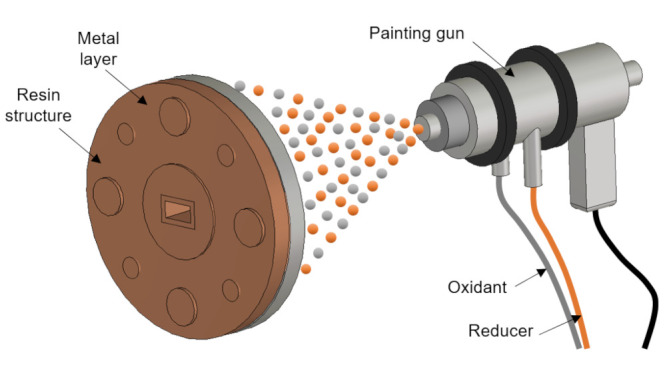
Spray metallization scheme developed by JetMetal [47].

On the other hand, another kind of plastic metallization process for millimeter band applications can be analyzed. The direct-platting technology of JetMetal [47] avoids some disadvantages of other plating processes, especially by providing significant cost reduction due to the short time required to apply it (i.e., only a couple of minutes). As can be observed in Figure 11, a compressed-air and double-nozzle painting gun is used to simultaneously spray a double solution of reducer (palladium free) and oxidant (composed of metallic silver ions Ag+) agents onto the 3D printed plastic device. The ratio of the reducing and oxidating agents forming the sprayed liquid film and their reaction conditions are properly controlled on the plastic surface of the material compounding the 3D printed device. In this manner, the silver metal coating is absorbed by the plastic, which is the key point of this process, avoiding the risk of crumbling and peeling observed in chemical platting. The main disadvantage of this metallization method, compared to the other methods mentioned, is that the spray must have direct access to the areas to be metallized. Therefore, this method cannot be applied to structures that have intricate topologies or that contain internal channels (unless it is possible to implement openings that allow access to them).

**Figure 12:**
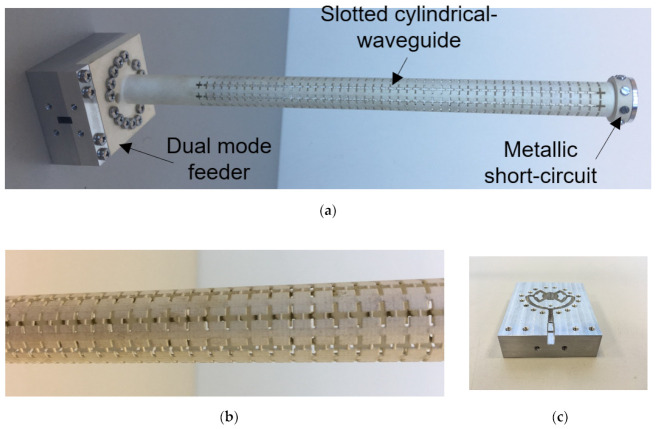
Photographs of the conformal array antenna manufactured by SLA + spray metallization: (**a**) whole antenna prototype; (**b**) slotted cylindrical waveguide detail; and (**c**) dual-mode feeder.

Several examples of antenna devices operating in the millimeter band, manufactured by using 3D SLA printing + JetMetal metalization coating, can be found in [48,49,50,51]. The metallization process, material composition, deposition time, and metallic layer thicknesses are extensively detailed in [50]. This work shows the comparison of a 77 GHz reference horn antenna fabricated by electroforming, SLA + copper electroplating, and SLA + silver spray metallization, demonstrating that the spray metallization method provides good results with a difference between simulated and measured gain of 0.6 dB. A tunable microwave device at Ku-band (a rotary vane attenuator) is also manufactured by the spray metallization technique in [51]. The authors successfully checked these 3D printing and post-metallization processes in a single band conformal slotted array [52], as well as in a dual-band and dual-polarization conformal slotted array for 5G applications [53], as shown in Figure 12a. In both cases, conical-beam antennas based on a slotted cylindrical waveguide path were designed. The latter was manufactured in resin material by SLA technology, then plated by metallic pulverization to experimentally validate the proposed antenna topology and the fabrication processes. The combination of these manufacturing processes led to optimized costs, precision, and reliability. Initially, the idea was to directly print the structure on a metal 3D printing process, such as DMLS. Nevertheless, the cross-slot inclusion in the cylindrical waveguide was incompatible with accurate manufacturing, although enquiries were made to several suppliers. In addition, this DMLS process is significantly more expensive than plastic SLA 3D printing. This is an important consideration, in addition to the considerable weight reduction, especially in a cellular system such as the next 5G mobile communication generation, in which massive deployment is required and the cost increase is a significant aspect.

The manufacturing processes involved in the different parts of the antenna presented in [52] were described. In the first instance, the cylindrical waveguide (including all slots) was manufactured with Accura Xtreme resin material and a 100 μm thick layer resolution, using a SLA 3D printing process performed by Protolabs [26].

Second, the 3D printed prototype was plated by the JetMetal sprayed metallization process [47], as shown in Figure 12b. In this context, the optimal metal thickness was significant. In both of the previously detailed processes (chemical Swissto12 or sprayed JetMetal coatings), the metal thickness could be controlled with the exposure time, set beyond the E-field penetration depth at the operation frequency band. At first sight, a silver coating thickness of just a few micrometers may seem brittle and prone to easy removal. In fact, the standard ECSS-Q-ST-70-71C Rev.1 (2019), followed by the European Space Agency (ESA), states that plated layers of less than 1 µm thickness tend to be porous, which may lead to corrosion. On the other hand, the same ESA standard reminds us that thick coatings can generate mechanical stresses and fairly high temperatures during their cure. In addition, thicker coatings require larger amounts of material, thus increasing costs. For this reason, coating thickness should be carefully chosen, taking into account mechanical, thermal, and electrical aspects.

Coating thicknesses in the order of microns are common in the space industry. As the abovementioned ESA standard states, aluminum surfaces are frequently treated with alodine, a conductive coating with a thickness of less than 1 µm. Other protective coatings to prevent electrostatic discharges and provide thermal relief can also have thicknesses lower than 1 µm [54]. Regarding the silver plating, which is usually employed to maximize electrical conductivity, thicknesses around these values are also often considered. The reflector of the Kepler Space Telescope is coated with a 0.5 micrometer silver layer [55], and some Ka-band waveguide devices have been post-processed with a 1–2 micrometer silver coating [56]. The latter have successfully undergone environmental tests, being exposed to severe humidity and thermal conditions or mechanical stress.

Other surface treatments can be applied to prevent corrosion and provide thermal relief. These treatments are usually in the form of paints, examples of which can be found in the standard ECSS-Q-ST-70-71C Rev.1, with thicknesses in the order of tens of microns. Thus, the 3D antenna prototype printed in plastic material was plated with a 2 μm silver coat by the spray metallization process developed by JetMetal.

Finally, some parts were mechanized in aluminum, following a traditional methodology based on computer numerical control (CNC). For example, the dual-mode feeder shown in Figure 12c, as well as the metallic short-circuit of the cylindrical waveguide, shown in Figure 12a, were both manufactured by CNC techniques. The only critical part in the assembly process of the 3D printed antenna prototype can be the screws fixing to the contact parts of the metallized device. The JetMetal process significantly reduces this risk, due to film absorption. In addition, the authors have tested the tightening of the screws several times in the same hole, without any significant degradation of the metallic coat. Nevertheless, if an extra-protection of these punctual areas is desired, metallic washers can be embedded inside the plastic to permit screw contact over them instead of over the plastic surface, as was implemented in a filter device for space applications manufactured by Swissto12 [57].

To demonstrate the strength of the manufacturing method described in this section, the experimental results of the conformal array shown in Figure 12 are presented. This antenna operates in a dual 5G band with dual polarization. In particular, the dual-mode feeder shown in Figure 12c has two input ports: port 1, implemented in a coaxial transition, and port 2, implemented in a rectangular waveguide transition. Port 1 generates the TM_01_ mode into the cylindrical waveguide, exciting the transversal slots. They are designed to operate in the n257 band (26–30 GHz). The slot arrangement around the cylindrical waveguide perimeter provides a uniform excitation of the slots, generating a linearly polarized ($\hat{\theta }$-polarization) conical-beam radiation pattern.

**Figure 13:**
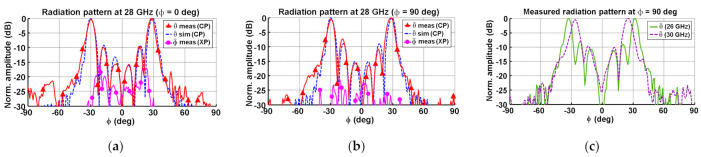
Conformal array antenna normalized radiation patterns: (**a**) simulated and measured ϕ = 0° patterns at 28 GHz; (**b**) simulated and measured ϕ = 90° patterns at 28 GHz; and (**c**) measured ϕ = 90° patterns at 26 and 30 GHz.

A comparison between the simulations (extracted from CST Microwave Studio) and the measurements is shown in Figure 13. In particular, the ϕ = 0° normalized radiation pattern at 28 GHz is shown in Figure 13a. A great concordance between the simulated pattern and the measured copolar (CP) radiation pattern was obtained, as well as a low cross-polar (XP) radiation. The difference between CP and XP maximum levels, denoted as cross-polar discrimination (XPD), was higher than 15 dB. In the case of the ϕ = 90° cutting plane (Figure 13b), a great similarity between simulation and measurements, as well as a XPD > 20 dB, was achieved. The similitude between CP radiation patterns, for both ϕ = 0° and ϕ = 90° cutting planes, demonstrated the omnidirectional performance of the conical-beam pattern. Finally, the experimental radiation patterns at 26 and 30 GHz are shown in Figure 13c. Considering the typical main beam scanning of the progressive-wave array antennas, the obtained results may be considered as stable in band.

Second, port 2 of the dual-mode feeder shown in Figure 12c was implemented in waveguide technology. A TE_10_–TE_01_ converter was used to transform the fundamental TE_10_ mode of the input rectangular waveguide into the non-fundamental TE_01_ mode of the cylindrical waveguide. This propagation mode produced an excitation of the longitudinal slots, which were designed to operate in the n260 band (37–40 GHz) [1]. Therefore, a linearly polarized ($\hat{\phi }$ -polarization) conical-beam radiation pattern was generated.

**Figure 14:**
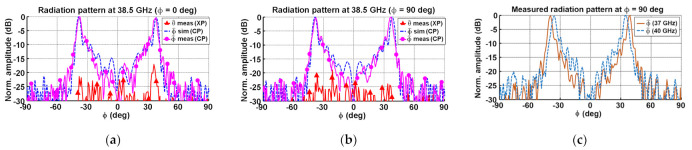
Conformal array antenna normalized radiation patterns: (**a**) simulated and measured ϕ = 0° patterns at 38.5 GHz; (**b**) simulated and measured ϕ = 90° patterns at 38.5 GHz; and (**c**) measured ϕ = 90° patterns at 37 and 40 GHz.

Figure 14a and Figure 14b present the CP and XP radiation patterns at 38.5 GHz for ϕ = 0° and ϕ = 90° cutting planes, respectively. The great concordance between simulations and measurements demonstrated the high quality of the manufacturing process. Moreover, the ϕ = 90° radiation patterns at 37 and 40 GHz are presented in Figure 14c, showing a notable stability in band.

Finally, the realized gain and the total radiation efficiency were evaluated experimentally. The realized gain was higher than 13.5 dBi for both frequency bands, while the total efficiency oscillated between 83% and 90%. Very slight differences between simulated and measured gains were obtained (around 0.3 dB), demonstrating excellent antenna surface finishing. Therefore, it can be concluded that the final prototype provides a high-performance in terms of gain and efficiency. Moreover, the proposed 3D-printed manufacturing process can be considered as a lightweight and low-cost solution for 5G high data rate communications.

Table 4 shows a comparison of different types of antennas fabricated with SLA to demonstrate the maturity that the technology has acquired during recent years. This fabrication method provides high-gain antenna array designs with efficiency levels above 90%, as shown in [58] and [59]. The technology is widely proven in Ku, K, and Ka bands. However, satisfactory work can also be found in higher bands, as shown in [60]. Some designs with polarization versatility, such as those shown in [53] or [58], can also be implemented with SLA technology obtaining high quality and good performance.

## 5. Discussion

In this work, some manufacturing methods applied to microwave antennas and devices in waveguide technology were analyzed. Table 5 summarizes the main advantages and limitations of every methodology discussed in the previous sections. It should be noted that the surface finish of SLA technology is usually quite good, while the DMLS technology provides a significant roughness. Although this roughness will be progressively reduced as the technology evolves, an effective conductivity reduction can be found for some high-frequency devices. On the other hand, high-precision machining will offer very high levels of conductivity and surface finish but will not allow the fabrication of devices with internal channels in a monolithic piece. This is a very important advantage of additive manufacturing technologies because the implementation of a prototype in several parts can produce some leakage effects (especially at higher frequencies). The average weight of the devices manufactured by conventional machining and DMLS technology is quite similar, but average weight is drastically reduced by SLA technology, as the prototypes are usually manufactured in plastic material. For the same reason, the cost of SLA technology is usually lower than that of alternatives. Regarding the level of technology maturity, it is obvious that high-precision machining is the most developed manufacturing method, while DMLS and SLA are under continuing evaluation. However, both are undergoing rapid evolution in recent years, due to the growth in the microwave field, so their performance is expected to improve in the coming years.

## 6. Conclusions

This article has presented an overview of different manufacturing techniques for state-of-the-art waveguide-based devices for the front-end of communication systems at the millimeter-wave band. Many types of manufacturing techniques have been developed during recent years, especially with the evolution of additive manufacturing, which has spread to all fields of engineering. Nevertheless, in the context of modern communication systems, there is not an optimum technique that can be used to build all devices. On the contrary, it is important to know which technique is most suitable for each device, and also to tailor the design of the device to the manufacturing technique that will be used. In fact, it is typical that different manufacturing techniques must be combined to build a single device.

The application of high-precision subtractive manufacturing techniques was illustrated with a monopulse antenna. Direct metal laser sintering was shown with a reconfigurable linear phased array antenna, which presented good performance despite the inherent roughness achieved by DMLS. Finally, devices combining stereolithography with metallization were shown. This approach can overcome certain roughness problems that appear in DMLS devices, while also producing lighter and less expensive devices.

## Figures and Tables

**Table 1:** Comparison of triple-radiation pattern monopulse antennas by high-precision subtractive machining.

Parameter	[22]	[33]
$f_{0}$ (GHz)	35	93
Sum Gain (dBi)	24	20
Diff Gain (dBi)	22	18
Sum Cxp (dB)	−21	N/A
Diff Cxp (dB)	−21	N/A
BW (%) @ 20 dB	2.3	3.0
Isolation (dB)	33	25

**Table 2:** Measured and desired phase shift (in degrees) produced by each of the phase shifters in the WRPS to scan the main lobe of the array to different directions $\theta_{0}$ (β is the necessary progressive phase shift for each scanning direction and $\beta_{i},\;i=1,..,4$ is the phase shift that each phase shifter must introduce).

		Measurements			Desired		
$\theta_{0}$	β	$\beta_{2}-\beta_{1}$	$\beta_{3}-\beta_{1}$	$\beta_{4}-\beta_{1}$	$\beta_{2}-\beta_{1}$	$\beta_{3}-\beta_{1}$	$\beta_{4}-\beta_{1}$
0	0	358.3	358.8	0.0	0	0	0
5	25.1	26.0	48.0	74.3	25.1	50.2	75.3
10	50.0	49.1	105.0	160.6	50.0	100.0	150.0
15	74.5	77.9	153.3	224.0	74.5	149.0	223.6
20	98.5	92.7	200.4	297.1	98.5	197.0	295.5
25	121.7	115.9	246.1	5.5	121.7	243.4	5.14

**Table 3:** Comparison of phased arrays with beam-steering capabilities.

Parameter	[36]	[37]	[38]	[39]
Nº of elements	4 × 1	4 × 4	4 × 1	20 × 1
$f_{0}$ (GHz)	17.0	28.0	5.5	9.4
Scanning range ( ∘ )	50	40	90	14
Power losses (dB)	0.46	8.50	1.50	0.73
Measured gain (dB)	12.0	10.7	11.0	19.1
BW @ 12 dB	23.5	9.0	12.8	4.3

**Table 4:** Comparison of high-gain antenna designs manufactured by SLA technology.

Parameter	[53]	[58]	[59]	[60]	[48]
Description	Conformal slotted array	Linear slotted array	Linear slotted array	Planar slotted array	Corrugated Plate Antenna
Polarization	Dual	Dual	Single	Single	Single
Frequency (GHz)	38.5	23.3	21.7	70.0	11.3
Efficiency (%)	85/89	87/92	95	71/89	56.6
Peak gain (dBi)	13.5/14	17.8/18.2	29.5	20.0	18.7
Beam shape	Conical	Pencil	Pencil	Pencil	Pencil

**Table 5:** Comparison between manufacturing technologies for millimeter-wave antennas.

	High-Precision Subtractive Machining	Direct Metal Laser Sintering	Stereolithography + Post-Metallization
Surface finish	Perfect	Poor	Good
Internal channel manufacturing	No	Yes	Yes
Effective conductivity	Very high	Medium	High
Cost	Medium	Medium	Low
Weight	Heavy	Heavy	Light
Maturity	High	Low	Medium
